# Use of an electromagnetic colonoscope to assess maneuvers associated with cecal intubation

**DOI:** 10.1186/1471-230X-9-24

**Published:** 2009-04-09

**Authors:** Russell I Heigh, John K DiBaise, James A Prechel, Billie J Horn, Sarah San Miguel, Evelyn G Heigh, Jonathan A Leighton, Cynthia J Edgelow, David E Fleischer

**Affiliations:** 1 Division of Gastroenterology, Mayo Clinic Arizona, 13400 East Shea Blvd, Scottsdale, Arizona 85259, USA; 2 Department of Kinesiology, College Of Liberal Arts and Sciences, Arizona State University 300 East University Drive, Tempe, Arizona 85287, USA

## Abstract

**Background:**

Safe and effective colonoscopy is aided by the use of endoscopic techniques and maneuvers (ETM) during the examination including patient repositioning, stiffening of the endoscope and abdominal pressure.

**Aim:**

To better understand the use and value of ETM during colonoscopy by using a device that allows real-time imaging of the colonoscope insertion shaft.

**Methods:**

The use of ETM during colonoscopy and their success was recorded. Experienced colonoscopists and endoscopy assistants used a commercially available electromagnetic (EM) transmitter and a special adult variable stiffness instrument with 12 embedded sensors to examine 46 patients. In 5 of these a special EM probe passed through the instrument channel of a standard pediatric variable stiffness colonoscope was used instead of the EM colonoscope.

**Results:**

Thirty-nine men and 7 women with a mean age of 64 years (range 33–90) were studied. The cecum was intubated in 93.5% (43/46). The mean time to reach the cecum was 10.6 minutes (range 3–25). ETM were used a total of 174 times in 41 of the patients to assist with cecal intubation. When ETM were required to reach the cecum, and the cecum was intubated, an average of 3.82 ETM/patient was used. While ETM were used most often when the tip of the colonoscope was in the left side of the colon (rectum 5.0%, sigmoid colon 20.7%, descending colon 5.0%, and splenic flexure 11.6%), when the instrument was in the transverse colon (14.8%), hepatic flexure (20.7%) and ascending colon (19.8%) the use of ETM was also required. When the colonoscope tip was in the transverse colon, hepatic flexure and ascending colon, ETM success rates were less (61.1%, 52.0%, and 41.7% respectively) compared to the left colon success rates (rectum 83.3%, sigmoid colon 84.0%, descending colon 100%, and splenic flexure 85.7%).

**Conclusion:**

The EM colonoscope allows imaging of the insertion shaft without fluoroscopy and is a useful device for evaluating the efficacy of ETM. ETM are important tools of the colonoscopist and are used most often in the left colon where they are most effective.

## Background

The performance of **s**afe and effective colonoscopy is aided by the use of endoscopic techniques and maneuvers (ETM) during the examination. Among the ETM commonly used are patient repositioning, stiffening of the colonoscope, and the application of abdominal pressure. The endoscopy unit at Mayo Clinic Arizona employs these techniques, teaches them to our trainees, endoscopy unit staff and others interested in the performance of colonoscopy [1,2]. An inability to actually see the problem impeding forward advancement of the colonoscope (e.g., looping, angulation) has limited the understanding of the benefits and limitations of the ETM. The recent commercial availability of an electromagnetic colonoscope and an accessory package was felt to be of potential use in this regard. Initial reports of use of an electromagnetic colonoscope sought to develop a means to view the instrument shaft in real-time without the need for fluoroscopy [3,4]. Electromagnetic coils to generate low strength magnetic fields and detected by sensors in the colonoscope insertion shaft were used and the colonoscope position was estimated based on a computer algorithm using triangulation principles. The prototype colonoscopes were subsequently refined by incorporating enhanced computing techniques to improve the accuracy, and refined computer graphics to improve usability [5]. In 2002, the Olympus Corporation introduced the first commercially-available, standardized electromagnetic colonoscope system. The aim of our study was to use this commercially available electromagnetic colonoscope to better understand the use of ETM in terms of when and why needed, and the ultimate success in allowing forward advancement of the colonoscope. This was done as part of a 6-week clinical evaluation of the instrument and accessories in our routine colonoscopy practice.

## Methods

An electromagnetic colonoscope that is United States Food and Drug Administration approved and available worldwide was loaned to our endoscopy by the Olympus Corporation for use in our clinical practice between 9/14/2007 and 11/8/2007. Data about its use were collected prospectively to determine how this instrument might fit in with, and possibly improve, our colonoscopy practice. The retrospective analysis of this data was approved by the Mayo Clinic Institutional Review Board.

### Instrument

An electromagnetic (EM) transmitter and a special adult variable stiffness instrument (outer diameter 13.2 mm, length 1680 mm, 140 degree view angle, and 3.7 mm instrument channel) with 12 embedded sensors (ScopeGuide (SG) Magnetic Endoscope Imaging System, CF-Q160DL, Olympus Corp. America, Center Valley, Pennsylvania) was used. The system consists of 2 main components: 1) Electromagnetic coils that generate a low level of energy are in a plate-like device positioned next to the patient, and 2) 12 sensor coils are built into the colonoscope shaft. A continuous real-time view of the colonoscope shaft is displayed both on a dedicated auxiliary monitor and on the main endoscopy monitor as an inserted picture next to the endoscopic image. The images can be viewed in two planes: lateral or anterior-posterior. An accessory catheter probe device that has sensors embedded in a cable that may be advanced through the working channel of a standard colonoscope also accompanies the system. The accessory catheter allows the shaft of any colonoscope to be visualized with this system. Additionally, an accessory marker sensor allows the position of the hands of an assistant providing abdominal pressure to be identified on the same screen.

### Patients

The ScopeGuide system was used when the endoscopist determined a patient was suitable for examination with an adult variable stiffness colonoscope. Patients with a pacemaker or implantable defibrillator were excluded. The first patient of a half-day endoscopy session was studied so that the regular endoscopic procedure schedule would remain unaffected by the trial. The EM accessory catheter probe was selected for use when the colonoscopist preferred to use a pediatric variable stiffness colonoscope to examine a patient. The usual clinical practice of the 3 colonoscopists is to use a pediatric variable stiffness colonoscope in most women, and in about half of the men referred for colonoscopy.

### Endoscopy Team

Three highly experienced colonoscopists (RIH, JKD, DEF) and three highly experienced endoscopy technicians (JAP, BJH, SSM) performed the examinations.

### Endoscopic Techniques and Maneuvers

While the colonoscope was being inserted the endoscopy nurse or observing endoscopy technical assistant recorded ETM and its success or failure. Instrument stiffening, patient position change, external abdominal pressure application, reasons for application, and colonoscope tip position during application were noted. When stiffening of the colonoscope was utilized, the instrument was adjusted from baseline minimum stiffness to maximum stiffness. Maneuver success was determined by the endoscopist at time it was used and was defined as the ability to further advance the colonoscope. Maneuver success or failure was recorded during the examination.

## Results

### Patient Information

A total of 46 patients were examined. Of these, 39 were men. The age range was 33–90 years with a mean of 63.6 years. The weight range was 52–110 kg with a mean of 83.1 kg.

### Colonoscopy Information

The cecum was reached in 43/46 patients (93.5%). The time required to reach the cecum and achieve cecal intubation was 3–25 minutes with a mean of 10.6 minutes. Of the 46 patients examined, 41 were examined with the dedicated electromagnetic colonoscope. In 5 patients the EM catheter probe with sensors was used along with a standard pediatric variable stiffness colonoscope. In these cases, the instrument shaft was imaged just as well as with the dedicated adult colonoscope with the built-in sensors; however, the use of this probe was ultimately abandoned due to its effect of limiting suction capability. All patients were American Society of Anesthesiology class I or II functional status, and received intravenous midazolam and meperidine or fentanyl under direction of the gastroenterologist to achieve moderate levels of sedation.

### Endoscopic Techniques and Maneuvers

Endoscopic techniques and maneuvers (ETM) were used during colonoscopy in 89.1% (41/46 patients). For all patients in the study, a total of 174 ETM were used. For the patients where the cecum was reached (43 patients), a total of 145 ETM were used, with a mean ETM per patient of 3.37 (145 ETM/43 patients). For the 38 patients who actually required ETM for successful scope advancement to the cecum (5 reached cecum without ETM), the ETM per patient was 3.82 (145 ETM/38 patients).

The reasons for use of ETM are presented in Table 1. The majority of the ETM were used when the colonoscope tip was in left side of the colon. Rectosigmoid angulation, sigmoid colon loop formation, angulation of the sigmoid colon, descending colon loop formation, splenic flexure loop formation and angulation of the splenic flexure accounted for 72.6% of reasons for ETM use.

**Table 1:** Indications for Endoscopic Techniques and Maneuvers

Rectosigmoid Angulation	3.3%
**Sigmoid Loop**	47.1%

**Sigmoid Angulation**	1.6%

**Descending Loop**	2.4%

**Splenic Flex Loop**	11.6%

**Splenic Flex Angulation**	6.6%

**Transverse (Trans) Loop**	9.0%

**Hepatic Flex Angulation**	9.9%

**Unspecified**	8.5%

The position of the colonoscope tip when ETM were required is presented in Table 2. While the colonoscope scope tip position was in the left colon or transverse colon the majority of the time when ETM where applied 59.5%, the colonoscope tip was at the hepatic flexure or ascending colon 40.5% of the time. Less success of ETM was noted when ETM were used when the colonoscope tip was in the transverse colon (61.1%), hepatic flexure (52%) and ascending colon (41.7%) compared to elsewhere in the colon.

**Table 2:** Position of the Colonoscope Tip and Success When Endoscopic Techniques and Maneuvers were Required

	Frequency	Success
**Rectum**	5.0%	83.3%

**Sigmoid**	20.7%	84.0%

**Descending**	5.0%	100.0%

**Splenic Flex**	11.6%	85.7%

**Transverse Loop**	14.8%	61.1%

**Hepatic Flex**	20.7%	52.0%

**Ascending**	19.8%	41.7%

**Figure 1:**
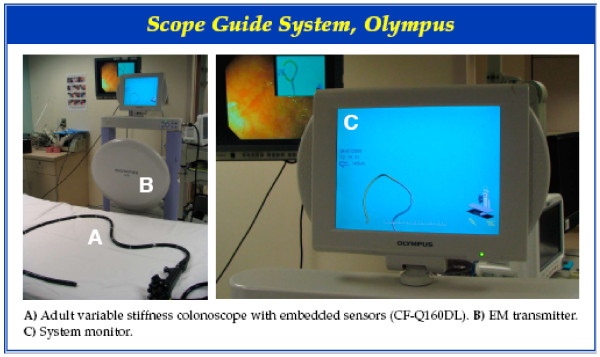
**Scope Guide System, Olympus**.

**Figure 2:**
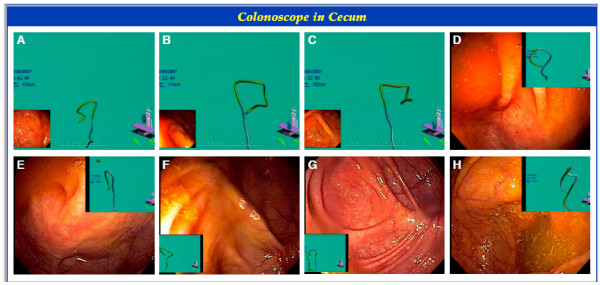
**Colonoscope in Cecum**. Different patients (n = 8) with the electromagnetic colonoscope tip in the cecum. Patients A, B, and C are monitor views of full frame electromagnetic colonoscope images, with picture-in-picture endoscopic images. Patients D, E, F, G, and H are full frame monitor views of endoscope image with electromagnetic colonoscope picture-in-picture images. Various orientation and display options are depicted.

**Figure 3:**
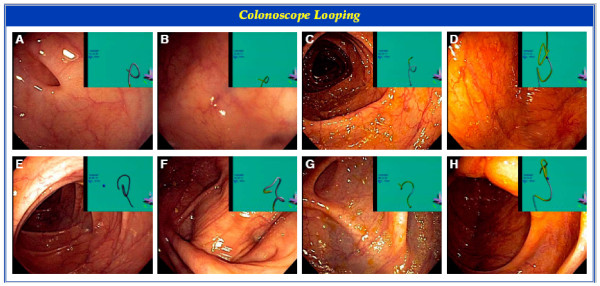
**Colonoscope Looping**. Different patients (n = 8) with looping at various colon positions depicted on the electromagnetic colonoscope picture-in-picture insert.

The success of the various Endoscopic Techniques and Maneuvers utilized (n = 174), is presented in Table 3. Patient repositioning, stiffening of the endoscope, and applying abdominal pressure had success rates of 73.7%, 69.2%, and 71.8% respectively. Figure 1 depicts the Olympus Scope Guide System. Figure 2 and Figure 3 demonstrate various views of the colonoscope in the cecum and the looping of the colonoscope.

**Table 3:** Frequency and Success of Different Endoscopic Techniques and Maneuvers (n = 174 ETMs)

	Frequency	Success
**Patient Repositioning**	28/38	73.7%

**Stiffening of Endoscope**	18/26	69.2%

**Abdominal Pressure (Varied types & sites)**	74/103	71.8%

**Unspecified**	4/7	57.1%

## Discussion

Our experience with a commercially available electromagnetic colonoscope demonstrates that the insertion shaft of the colonoscope can indeed be reliably visualized without the need for fluoroscopy. This ability may shed light on the effectiveness of individual endoscopic techniques and maneuvers to facilitate cecal intubation and may improve the teaching of colonoscopy to trainees and endoscopy assistants.

We have found that a commercially-available electromagnetic colonoscope can be used to reliably assess the position and location of the colonoscope during an examination and may be useful to assess the efficacy of ETM. In the patients evaluated, 89.1% required the assistance of ETM to reach the cecum, and when successful, an average of 3.82 ETM per patient were required. The majority of the EMT were used because of colonoscope looping when the colonoscope was in the left side of the colon; however, transverse colon looping and hepatic flexure angulations were also relatively common reasons for the need to use these techniques. Of particular interest, when the colonoscope tip was in the transverse colon, hepatic flexure, and ascending colon, the success rates of ETM were less than when in the left colon.

Visualization of the insertion shaft position has been studied by others with mixed results regarding its clinical utility. In a study involving 100 patients in which a prototype electromagnetic colonoscope was used, looping occurred in 91%; sigmoid looping (78%) and deep transverse looping (34%) were most common [6]. Interestingly, most loops were incorrectly diagnosed (position or type) by the colonoscopist (69%) without use of the information gained from the imaging device. With regards to ETM use, abdominal pressure was effective in producing forward movement of the colonoscope only 54/154 times (37%), whereas position change was effective 95/144 times (66%). Sigmoid pressure was ineffective due to hand misplacement in 36%, incorrect diagnosis of looping or inaccessible looping in 52%. Transverse colon pressure was often unsuccessful due to under recognized or unappreciated sigmoid looping in 86%. In another study of the use of the electromagnetic colonoscope involving 78 patients, looping occurred in 33% of the cases and led to the application of abdominal pressure and early position changes that assisted the completion of the examination [7]. Importantly, the device was also found to be accurate in estimating lesion location and was suggested to be helpful in difficult examinations. More recently, in a relatively large study, patients undergoing unsedated colonoscopy were shown to have a significantly higher cecal intubation rate when the EM scope was used compared to the same instrument with out EM shaft visualization (90% vs. 74%; *P *< 001) [8].

The EM colonoscope has also been used to study the utility of the variable stiffness colonoscope [9]. In 257 patients studied, cecal intubation times were shorter and fewer ancillary maneuvers were required when a variable stiffness colonoscope was used. Maximal effectiveness of the variable stiffness colonoscope occurred when the device was stiffened after the sigmoid colon was negotiated and the colonoscope was straightened in the proximal colon. These investigators went on to demonstrate that the EM scope allowed accurate loop assessment and scope straightening during colonoscopy, but did not result in a reduction of sedation dosage during colonoscopy when the overall medication doses were small [10].

In contrast to the above reports, other studies have failed to show a clinical benefit of the electromagnetic colonoscope outside of a training setting [11]. In a 120 patient study involving experienced endoscopists, the electromagnetic colonoscope offered no performance improvement. The only benefit noted was in localizing lesions and it was felt that the instrument was mainly of benefit in localizing small tumors before colorectal surgery. The early mixed evaluations of the use of an electromagnetic colonoscope prompted an editorialist to describe the instrument as a "GPS Device for the Colon" in the sense that expert navigators may never need help with directions on well travelled routes; however, inexperienced drivers may benefit from the use of a navigation system [12].

Although our study is limited by examining only a small and select subset of our large colonoscopy practice (approximately 6,500 colonoscopies year), it is clear that the technology is able to accurately visualize the colonoscope configuration and positions, and may be of use in understanding ETM used in achieving cecal intubation during colonoscopy.

## Conclusion

The electromagnetic colonoscope can provide reliable information about the position of the colonoscope insertion shaft and may be helpful in understanding the usefulness of endoscopic maneuvers and techniques required to achieve cecal intubation. It is conceivable that with an appropriate study design, the instrument may be used to study when each specific ETM, either alone or in combination, may assist cecal intubation. Furthermore, while the use of this instrument by experienced colonoscopists may not be necessary with sedated colonoscopy practice, it may have an application in unsedated colonoscopy and in training physicians to perform colonoscopy or allied healthcare members to assist in colonoscopy examinations. Additional uses of the electromagnetic colonoscope and the technology behind it may emerge as the ability to visualize the insertion shaft and scope tip without fluoroscopy becomes more widely recognized. For example, rapidly evolving endoscopic techniques like enteroscopy might be enhanced by incorporating electromagnetic imaging technology into endoscope design.

## Competing interests

RIH: Previous research grant support from Olympus for study of video capsule small bowel imaging device, 2005–2006. JAL: Previous research grant support from Olympus for study of video capsule small bowel imaging device, 2005–2006. DEF: Previous research grant support from Olympus for study of video capsule small bowel imaging device, 2005–2006. JKD, JAP, JBH, SSM, EGH, CGE: No competing interests.

## Authors' contributions

RIH, JAP, and DEF conceived the study, participated in its design and coordination and helped draft the manuscript. JKD, BJH, SSM, JAL, and CGE participated in study design and helped draft the manuscript, EGH performed the data analysis, statistical analysis, and helped draft the manuscript. All authors read and approved the final manuscript.

## Pre-publication history

The pre-publication history for this paper can be accessed here:

http://www.biomedcentral.com/1471-230X/9/24/prepub
